# An Ultrasonic-Capacitive System for Online Characterization of Fuel Oils in Thermal Power Plants

**DOI:** 10.3390/s21237979

**Published:** 2021-11-29

**Authors:** Mateus Mendes Campos, Luiz Eduardo Borges-da-Silva, Daniel de Almeida Arantes, Carlos Eduardo Teixeira, Erik Leandro Bonaldi, Germano Lambert-Torres, Ronny Francis Ribeiro Junior, Gabriel Pedro Krupa, Wilson Cesar Sant’Ana, Levy Ely Lacerda Oliveira, Renato Guth de Paiva

**Affiliations:** 1 Gnarus Institute, R&D Department, Itajuba 37500-052, MG, Brazil; mateusmcampos@unifei.edu.br (M.M.C.); carlos.teixeira@institutognarus.com.br (C.E.T.); erik@institutognarus.com.br (E.L.B.); germano@institutognarus.com.br (G.L.-T.); ronny@institutognarus.com.br (R.F.R.J.); gabriel@institutognarus.com.br (G.P.K.); levy@institutognarus.com.br (L.E.L.O.); 2 Institute of Engineering Systems and Information Technology, Itajuba Federal University, Pro-Reitoria de Pesquisa e Pos-Graduacao (PRPPG), Itajuba 37500-903, MG, Brazil; leborges@unifei.edu.br (L.E.B.-d.-S.); daniel_arantes@unifei.edu.br (D.d.A.A.); 3 Norte Energia S.A., Vitoria do Xingu 68383-000, PA, Brazil; renatopaiva@norteenergiasa.com.br

**Keywords:** heat value, heavy fuel oil, thermal power plant, ultrasonic-capacitive system, water content

## Abstract

This paper presents a ultrasonic-capacitive system for online analysis of the quality of fuel oils (FO), which are widely used to produce electric energy in Thermal Power Plants (TPP) due to their elevated heating value. The heating value, in turn, is linked to the quality of the fuel (i.e., the density and the amount of contaminants, such as water). Therefore, the analysis of the quality is of great importance for TPPs, either in order to avoid a decrease in generated power or in order to avoid damage to the TPP equipment. The proposed system is composed of two main strategies: a capacitive system (in order to estimate the water content in the fuel) and an ultrasonic system (in order to estimate the density). The conjunction of the two strategies is used in order to estimate the heating value of the fuel, online, as it passes through the pipeline and is an important tool for the TPP in order to detect counterfeit fuel. In addition, the ultrasonic system allows the estimation of the flow rate through the pipeline, hence estimating the amount of oil transferred and obtaining the total mass transferred as a feature of the system. Experimental results are provided for both sensors installed in a TPP in Brazil.

## 1. Introduction

The Brazilian electrical energy mix, since the 1990s, has shown a strong growth in Thermal Power Plants (TPP) based on fossil fuels [1]. According with the Brazilian regulatory agency for energy (ANEEL) [2], the granted electrical power to fossil fuels is approximately 35 GW (representing about 16% of the Brazilian energy mix). Among the fossil fuels, fuel oils (FOs, which include heavy and light fuel oils) represent a share of 23% (around 8 GW).

The efficiency of power generation in a TPP is linked with the quality of the FOs, besides the fact that oils with contaminants may induce to long-term damage to the equipment in the TPP. The most common contaminant is water. The process of contamination might be unintentional (due the processes involved in extraction and storage)—however, it might also be due to counterfeit. The counterfeit/adulteration of a high-quality fuel with some other substance of less quality and cheaper economical value is a common (and deceptive) practice around the world, as presented in scientific reports from Bangladesh [3], Ghana [4], India [5], and Tanzania [6]. In these aforementioned references, gasoline or diesel is mixed with subsidized kerosene or lubricants and affect automotive engines. In the case of the Brazilian TPPs, the fuel oils are usually loaded from tank trucks. These trucks are filled with an agreed amount of oil at a distribution center or refinery. During the transportation, it is reported [7] that some truck drivers remove a small quantity of oil (in order to sell it in the black market) and replace this amount with water.

The literature presents some methods to detect damage on the internal combustion engines that convert the chemical energy of the fuels into rotational mechanical energy at their crankshafts (which are tied to synchronous generators in order to produce electrical energy). Such methods may rely on the direct measurement of internal parameters of the engine (such as pressure and temperature at different parts of the engine) [8,9] or even the use of voltage and current signals of the synchronous generator [10] in order to indirectly detect mechanical issues within the engine. However, these methods cannot test the fuel quality, which may cause a problem that will only be manifested in the long term.

In order to check the quality of their fuels, the Brazilian TPPs perform laboratory analyses (which follow the standards ASTM D95 [11] for water content, ASTM D1298 [12] for density, and ASTM D4868 [13] for heat value) on samples. However, the samples taken are only a small fraction of the total amount of fuel that is loaded to the TPP pipelines. Moreover, in the case of TPPs located far from the large centers (which is a common scenario in the North and Northeast regions of Brazil), the laboratory tests are not even performed (due to lack of certified laboratories nearby). Hence, the use of online methods for the estimation of fuel quality (especially heavy fuel oils—HFO) is a general demand from the TPP shareholders.

### 1.1. Literature Review

#### 1.1.1. Online Measurement of Water Content in Fuel Oils

The work of Han et al. [14] presents a method based on differential pressure. Its advantage is that it this is a simple (and low cost) method, requiring only a differential pressure transducer. However, the accuracy of this method for estimating water content is strongly dependent on the difference in the specific mass of fuel oil and water.

The literature also presents methods based on absorption of electromagnetic waves, such as infrared (as presented by Al-Saiyed et al. [15]) and gamma ray (as presented by Scheers [16] and Johansen and Tjugum [17]). The main advantage of methods based on electromagnetic waves is the frequency range that can be used, whose wavelengths reach molecular levels, detecting the fraction of water in molecules as well as other contaminants, sulfur, vanadium, etc. However, these techniques are more common for laboratory applications that use small samples of fuel oils to be analyzed and obtain their characteristics. Considering online models, these techniques are limited to small ducts (from mm to few cm) [18], which differs from the applications proposed in the work (where the ducts are larger than 3${}^{\prime \prime }$). Another drawback of these techniques is the cost of processing.

The works of Gregory and Clarke [19] and Makeev et al. [20] present a method based on microwaves. The main advantage is the possibility to measure the whole range of water fractions (from 0% to 100%, with an extra advantage at the precision at lower fractions). However, this technology has a high initial cost in relation to the others, and it is also sensitive to variations in salinity in the range of higher fractions of water.

The work of Teixeira et al. [21] presents a method based on ultrasound. The advantage of this method is that it allows the gathering of information online, adaptable to conduits with larger diameters. However, the disadvantages are a relatively high cost and also the need for a cumbersome calibration process in order to classify water percentages.

Another technology for the estimation of the water content is the measurement of the capacitance of the emulsion. The fluid being measured acts as the dielectric of the sensor, making it possible to measure the electrical permittivity of the fluid and, consequently, the capacitance of the sensor–fluid system. The percentage of water in oil can be calculated based on a predictable relationship in the electrical properties of the emulsions. Hammer et al. [22] proposed a non-intrusive capacitive sensor, capable of measuring water contents in the range between 0% and 80%. Shuller et al. [23] proposed a measurement principle of the dielectric properties of the emulsion based on a single electrode excited by an oscillator (in the range of Mega Hertz). Libert et al. [24,25] presented a system based on capacitive sensors installed at the internal wall of the pipes.

Systems such as EIT (Electrical Impedance Tomography) or ECT (Electrical Capacitance Tomography) are techniques widely researched and disseminated in the literature [26,27,28] with great advances in the field of flow measurement and physical– characterization of multiphase fluids. The advantages found in these types of technique are mainly focused on the potential to be applied in multiphase fluids, to present the flow image in the penstock cross section and the possibility to be used on flow conditions unfavorable for other methods [26,27,28]. The disadvantages, however, are linked to the development of more consolidated techniques for commercial application, in addition to the complexity of electronics [26].

The capacitive sensor used in this current work was proposed by the authors of [29] and is based on paralleled disks, which are immersed in the emulsion inside the pipeline. The advantage of the system proposed in [29] is the electronic topology based on a Howland current source, capable of eliminating parasitic effects caused by influences from cables, connectors, and constructive characteristics of the capacitive sensor that affect the capacitance measurement. In addition, as will be seen in Section 2.2, the sensor is based on several disks in order to perform a paralleled association of the capacitances, which increases the total capacitance of the sensor. The fluid to be inspected fills the spaces between each pair of disks, forming small associated capacitors. Higher capacitance values allow it to have greater precision in measurements, as their proportion to the parasitic elements (such as those from cables, traces of plates and electronic parts) is greater, thus reducing the magnitude of errors caused by them.

#### 1.1.2. Online Measurement of Density in Fuel Oils

The literature presents several methods for measuring the density: many methods are based on direct physical relationships and others are based on indirect parameter determinations [30]. Most of the established methods have limitations inherent to the system, which often result in application restrictions in the sensing implementation (physical process limitations such as tube diameter, limitations in process deviations, limitation in the flow velocity of the fluid to be monitored, and others) [31]. In other cases, the presence of bubbles, particles, or incrustations may cause problems in specific mass measurements. Methods involving ultrasonic waves appear as alternatives to replace the standard methods, where these cannot be applied [32]. The most widespread ultrasonic techniques for measurement of density are based on three different principles: speed of sound in the propagation medium, acoustic impedance, and propagation on waveguides.

Waveguide approaches generally use time variations in the propagation of ultrasonic waves on a transmission line immersed in the measurement medium [33].

The acoustic impedance method approach is based on the determination of the reflection and transmission coefficient and the transmission coefficients of flat ultrasonic waves that propagate through different media [32].

Considering the determination of the density of any medium just by measuring the speed of sound, Swoboda et al. [34] used measurements of speed of sound and temperature in a sulfuric acid solution and found that the assessment of density was possible over a relative range greater than 1.10–1.30 and over a temperature range greater than 10 ${}^{\circ }$C to 50 ${}^{\circ }$C. The density and speed of sound are quantities that vary with temperature and pressure [35]. For determination of density in controlled systems, where there is little variation in temperature, pressure, and knowledge of the compressibility of the medium to be measured, the application of this concept is quite interesting, as it reduces the complexity of electronics in measuring the speed of sound and, by consequence, in the calculation of the density.

### 1.2. Aim and Objectives

The proposition of this work is to present a methodology for measurement of the quality of fuel oils based on two different strategies (capacitive and ultrasonic), in order to estimate the water content and heating values. The methodology is applied in an ultrasonic-capacitive hybrid on-line measurement system, which analyzes the fuel oil constantly throughout the entire fueling process. The advantage of this system is that it estimates the fuel oil quality parameters in real time, unlike the methods currently applied in Brazilian TPPs, which are based on laboratory analysis of small samples of fuel oil. However, laboratory results are not instantaneous, and some TPPs in Brazil do not even have certified laboratories nearby. Hence, the proposition of this paper allows for an immediate estimation of the fuel quality, while the fuel oil is flowing through the pipelines.

The originality of this paper lies precisely in the application of the proposed system at the loading bays of thermoelectric power plants for online monitoring of fuel oil used for energy generation.

### 1.3. Outline of the Paper

The paper is organized as follows: Section 2 presents the fundamental concepts about the two main strategies (capacitive and ultrasonic) and its correlation in order to infer about the fuel quality. Section 3 presents the experimental results obtained with the prototype installed in a Brazilian power plant. Finally, Section 4 presents the main conclusions of the work and some opportunities for future research.

## 2. Materials and Methods

**Figure 1:**
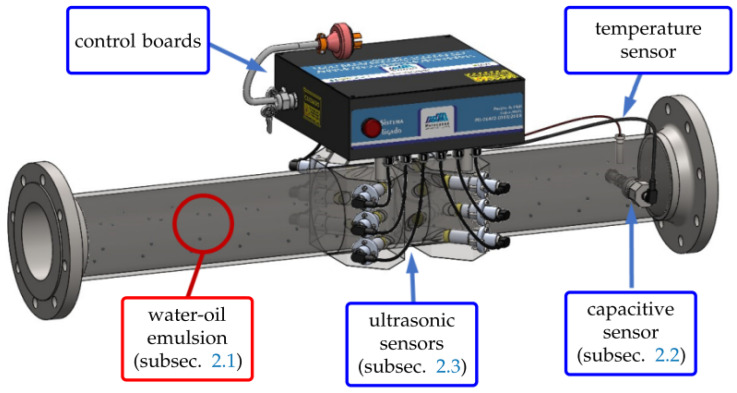
Conceptual 3D drawing of the proposed system.

Figure 1 presents the conceptual drawing of the proposed system. This system must be installed in a section of the pipeline between the TPP fuel storage and the point where the tank truck discharges the oil fuel. Section 2.1 briefly discusses the main characteristics of an emulsion of water-oil. As the emulsion passes through the pipe, the proposed capacitive sensor (described in detail in Section 2.2) estimates the water content of the emulsion, and the proposed ultrasonic sensors (described in detail in Section 2.3) estimate the density and the flow rate. The box indicated as “control boards” contains an FPGA board that controls and receives data sent from the capacitive system control board, ultrasonic system control board, and, also, from the temperature sensor. Through ethernet, the FPGA makes the data available to a remote analysis software, where the information of the aforementioned sensors is used to estimate the water content, the density and the heating value (hence, the quality) of the oil fuel. Section 2.4 presents the proposed procedure to estimate the heating value.

### 2.1. Characteristics of Fuel Oils

Fuel oils (FO) can be categorized in relation to viscosity as Light Fuel Oils (LFO) and Heavy Fuel Oils (HFO). FOs also have several other parameters that are used in their characterization—three of them, particularly in this work, are of special interest:

- **Water content** is probably the main contaminant of fuel oils, since contamination can occur in all parts of the production process, from the moment of extraction of crude oil to the moment of sending it to the TPPs [21]. The lower the water-in-oil content, the better the oil quality for power generation. The Resolution number 3 of the Brazilian National Agency of Petroleum, Natural Gas and Biofuels (ANP) stipulates a maximum of 2% water-in-oil content [36].
- **Density** is the ratio of the mass of oil to its volume. The density of the FO is an important property, which directly influences its combustion power and in the energy production, as well as in the removal of contaminants in centrifuges [37]. Density can be used to define other intrinsic characteristics of FO, such as the heat of combustion.
- **Heating Value** shows the amount of energy that could be obtained from fuel combustion. It is, usually defined in terms of a Higher Heating Value (HHV, or Gross Heat of Combustion (GHC)) and a Lower Heating Value (LHV, or Net Heat of Combustion (NHC)). The difference between the two is that HHV includes the latent heat of evaporation from the water vapor formed during combustion, while the LHV considers the loss of the latent heat needed to evaporate the water formed during combustion [38,39]. The LHV is the indicator used by Brazilian TPPs in order to estimate the generation capacity [36] and is the one that is going to be estimated in Section 2.4.

### 2.2. Estimation of Water Content through Capacitance

The estimation of water content in fuel oils is a welcomed measure in the power generation industry, as the presence of this contaminant reduces the efficiency of energy production. Moreover, the presence of water in fuel oil tends to be corrosive to system equipment, also causing environmental problems. Still, from an economic point of view, water has no economic value for energy generation.

**Figure 2:**
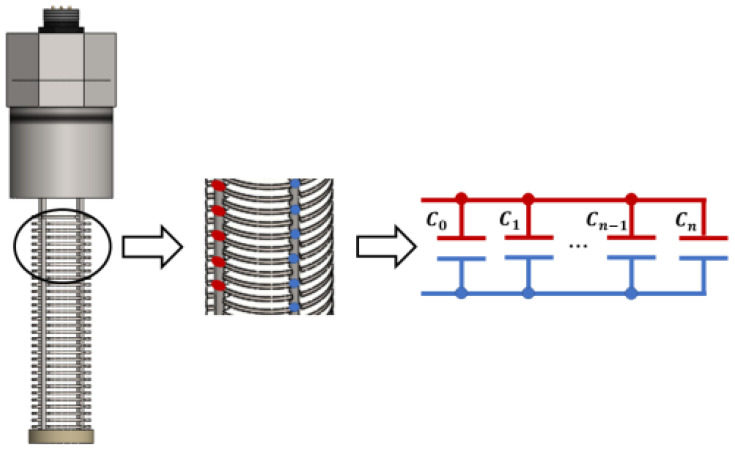
Proposed capacitive sensor and its equivalent circuit.

The authors proposed in [29] a capacitive sensor based on paralleled disk, as presented in Figure 2. As the sensor is immersed into the emulsion, the fluid act as the dielectric of an array of capacitors. The importance of having an array of paralleled capacitors is that higher values of capacitance increase the precision of the measurements, as the parasitic elements (from the cables, circuit boards, etc.) have less influence on the process. In addition, the proposed circuit uses a Howland current source topology, with enhancements that allow its continuous oscillating operation and easy adjustment of output current and frequency.

**Figure 3:**
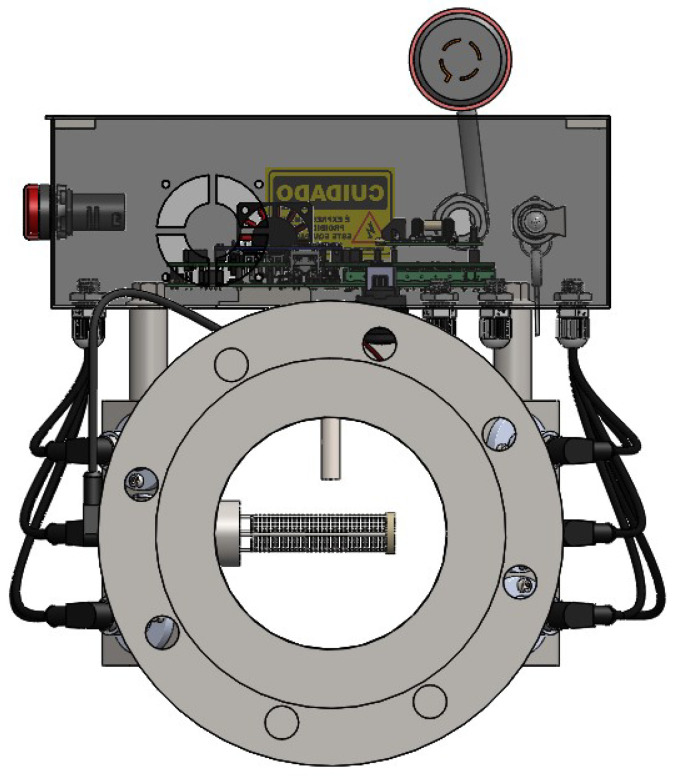
Proposed capacitive sensor installed in a transversal section of the pipeline.

The sensor must be installed in a transversal section of the pipeline, as presented in Figure 3. Its sensitive region is limited to the metallic disks that will be filled with the fluid. The length of this sensitive region is 8 cm.

The permittivity of water is approximately 40 times greater than the permittivity of the fuel oil [40,41]—hence, the greater the water content in the emulsion, the greater its measured capacitance will be.

In order to estimate the water content from the measured capacitance, a calibration procedure is performed, based on the capacitances of four emulsion samples with known water percentages (measured with a Karl Fischer laboratory equipment). (The calibration procedure must be performed for each different sensor, as there might occur some mechanical differences (such as the diameter of the metallic disks and the spaces between them) from one sensor to another.) Table 1 presents the data obtained in the calibration of the prototype of the capacitive sensor (used in the experimental results of Section 3). The left column contains the water concentrations (obtained in a Karl Fischer laboratory test) of the four fuel samples (prepared with pure HFO mixed with different amounts of water), and the right column contains the respective capacitances measured with the sensor.

Through a linear interpolation of the data of Table 1, Equation (1) is obtained. This equation relates a given measurement of capacitance (in pF) to the water content (in %).

(1) $$f_{water\%}=0.0087\cdot C_{pF}-2.0712\;,$$

where $C_{pF}$ is the measured capacitance value (in pF) and $f_{water\%}$ is the estimated water content (in%).

### 2.3. Estimation of Density and Flow through Ultrasonic System

#### 2.3.1. Density

According to [42,43], ultrasonic methods can determine the density $\rho_{u}$ of a fluid through the use of the Newton–Laplace Equation (2) and measurement of the speed of sound in that medium.

(2) $$\rho_{u}=\frac{1}{\kappa_{s}\cdot c^{2}}=\frac{B}{c^{2}}\;,$$

where $\kappa_{s}$ is the isentropic (adiabatic) compressibility of the fluid and *c* is the speed of sound at this fluid. Usually, the compressibility is also expressed in terms of its reciprocal, i.e., the bulk modulus $B=1/\kappa_{s}$ [32,44].

The speed of sound can be obtained either through a pulse-echo system [45] or through a system of two transducers (emitter–receiver) [46,47]. Both principles are based on the measurement of the wave propagation time in the fluid, differing only in the number of ultrasonic transducers used.

In case of a pulse-echo system, the basic principle involves the use of an ultrasonic transducer that outputs a very short pulsed signal of the proper ultrasonic frequency. The ultrasonic wave travels through the propagation medium to the other side, where a reflector material is positioned. Hence, there is a reflection of this ultrasonic wave, causing a large part of the energy of the ultrasonic wave to return through the medium to the transducer that emitted it.

In case of an emitter–receiver system, there are two transducers positioned on separate sides forming an acoustic trajectory between them. While one ultrasonic transducer emits, the other receives the ultrasonic signal that has propagated through the measurement medium.

**Figure 4:**
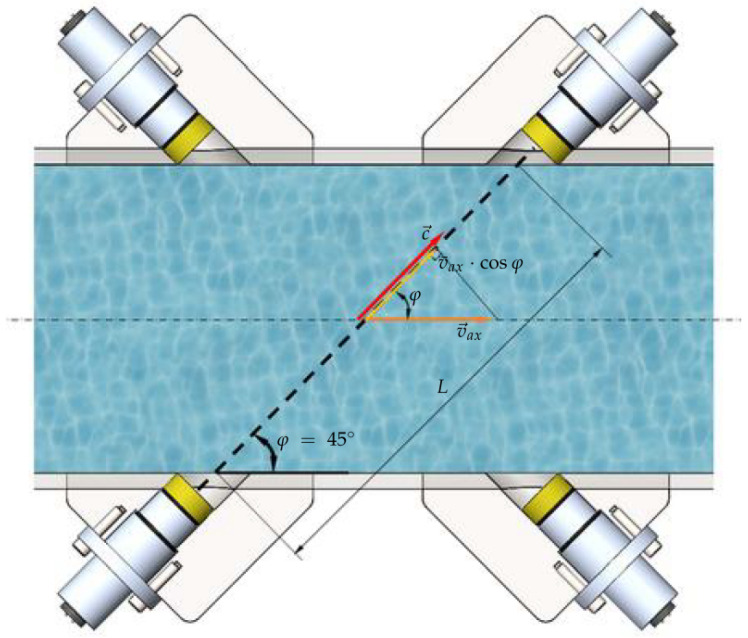
Measurement of time-of-flight in a conduit using multiple acoustic trajectories of ultrasonic transducers.

In the proposed system, multiple acoustic trajectories are used, as illustrated in Figure 4. In the figure, two transducers are opposed at the conduit and form an acoustic trajectory of length *L* and angle φ with the conduit wall. During transmission, the ultrasonic pulse that travels in favor of the fluid flow through the conduit (in downstream direction) travels the distance *L* in a time period $t_{down}$ smaller than the pulse that travels in the opposite direction (against the fluid flow, in upstream direction) in a time $t_{up}$.

Considering that, in Figure 4, ${\vec{v}}_{ax}$ is the axial velocity of the fluid flowing through the conduit (whose amplitude has a value of ${\bar{v}}_{ax}$), its projection on the trajectory *L* (at an angle φ with the flow direction) is given by ${\bar{v}}_{ax}\cdot \cos\varphi$. Hence, whenever there is a fluid flowing through the conduit, the speed of the ultrasonic pulses downstream ($v_{down}$) and upstream ($v_{up}$) can be calculated as (3) and (4), respectively.

(3) $$v_{down}=c+{\bar{v}}_{ax}\cdot \cos\varphi \;,$$

(4) $$v_{up}=c-{\bar{v}}_{ax}\cdot \cos\varphi \;.$$

In addition, the propagation times of the ultrasonic pulses downstream ($t_{down}$) and upstream ($t_{up}$) can be calculated as (5) and (6), respectively.

(5) $$t_{down}=\frac{L}{c+{\bar{v}}_{ax}\cdot \cos\varphi }\;,$$

(6) $$t_{up}=\frac{L}{c-{\bar{v}}_{ax}\cdot \cos\varphi }\;.$$

Combining Equations (5) and (6), it can be shown that the speed of ultrasonic wave *c* can be obtained as a function of the downstream and upstream travel times as per Equation (7).

(7) $$c=\frac{L}{2}\cdot \frac{t_{up}+t_{down}}{t_{up}\cdot t_{down}}\;.$$

As presented in Figure 1, the ultrasonic system has three layers of planes equivalent to Figure 4, and each layer has two crossed ultrasonic trajectories. Hence, Equation (7) can be modified to (8), in order to obtain an average velocity $\bar{c}$ considering each of the six trajectories $L_{i}$ and each of the six pairs of downstream and upstream times ($t_{down,i}$ and $t_{up,i}$, respectively).

(8) $$\bar{c}=\frac{1}{6}\sum_{i=1}^{6}\frac{L_{i}}{2}\cdot \frac{t_{up,i}+t_{down,i}}{t_{up,i}\cdot t_{down,i}}\;,$$

where *i* is an index corresponding to one of the six trajectories.

**Figure 5:**
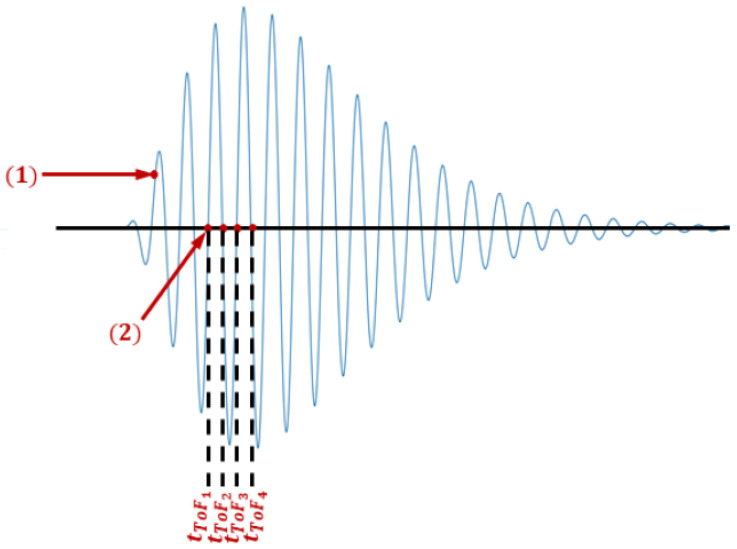
Measurement of the transit time through multiple zero crossings.

The technique used to measure the ultrasonic wave propagation times is based on the zero-crossing detection [48] of the pulses at the receivers. Figure 5 presents the detection of the zero-crossing of an ultrasonic wave. After the transmission of the ultrasonic pulse, the FPGA starts counting the time of several zero-crossings at the receiver. In order to avoid any initial transient, the first cycles are skipped. This is performed by detecting a pre-defined threshold (indicated by (1) in the figure). After the threshold, more three zero-crossings are skipped (until the first valid zero-crossing, indicated by (2) at the figure). Then the time from the starting of the pulse until next *M* number of zero-crossings ($t_{ToF1}$ until $t_{ToFM}$) are computed. The average time of the valid zero crossings is calculated (recursively) using Equation (9).

(9) $${\bar{t}}_{ToF}=\frac{1}{M}\cdot \left(t_{ToF1}+\sum_{j=2}^{M}\left[t_{ToFj}-\left(j-1\right)\cdot {\bar{t}}_{c/2}\right]\right)\;,$$

where ${\bar{t}}_{ToF}$ is the average of the zero crossing times. It is important to note that ${\bar{t}}_{ToF}$ can be any of the $t_{up,i}$ or $t_{down,i}$ times in Equation (8). Furthermore, ${\bar{t}}_{c/2}$ is the average time of the half cycle of oscillation of the signal, and is calculated with (10).

(10) $${\bar{t}}_{c/2}=\frac{1}{M-1}\cdot \sum_{j=1}^{M-1}\left[t_{ToFj+1}-t_{ToFj}\right]\;.$$

Finally, the average speed of the ultrasound waves (calculated as (8)) at the fluid is used in Equation (2) in order to obtain the density $\rho_{u}$ of the fluid.

#### 2.3.2. Flow Rate

According with [49,50], the flow rate $Q_{v}$ using multiple acoustic trajectories arranged at different heights in the cross section of the conduit can be calculated as (11). This calculation performs a numerical integration of *N* trajectories arranged in distinct heights in the cross section of the penstock. The most used integration methods [51] are: Gauss–Legendre (for rectangular sections) and Gauss–Jacobi (for circular sections). The OWICS (Optimal Weighted Integration, for circular sections) and the OWIRS (Optimal Weighted Integration for rectangular sections) [48] can also be highlighted. What differentiates the methods are the speed profiles [49,52], whereas the Gauss–Legendre and the Gauss–Jacobi propose uniform speed profiles [51].

(11) $$Q_{v}=\frac{D}{2}\cdot \sum_{i=1}^{N}w_{i}\cdot {\bar{v}}_{ax,i}\left(z_{i}\right)\cdot L_{i}\cdot \sin\left(\varphi_{i}\right)\;,$$

where $z_{i}$ is the position of the trajectory in relation to the cross section of the conduit. ${\bar{v}}_{ax,i}\left(z_{i}\right)$ is the average axial speed along trajectory *i*, calculated according to Equation (12). $w_{i}$ are weighting coefficients (which depend on the number of trajectories and on the integration technique in use, according to Equation (13)). *D* is the dimension of the parallel conduit for the intersection of two acoustic planes (i.e., the diameter of a circular section conduit). *N* is the total number of acoustic trajectories in a measurement plane. $L_{i}$ is the distance (length) of the acoustic trajectory *i*, and $\varphi_{i}$ is the angle between the acoustic trajectories.

(12) $${\bar{v}}_{ax,i}\left(z_{i}\right)=\frac{L_{i}}{2\cdot \cos(\varphi_{i})}\cdot \frac{t_{up}-t_{down}}{t_{up}\cdot t_{down}}\;,$$

where $t_{up}$ is the measured propagation time upstream and $t_{down}$ is the measured propagation time downstream. Their difference is known as $\Delta_{t}=t_{up}-t_{down}$.

According to [49,51], the weighting coefficients can be calculated as (13).

(13) $$w_{i}=\frac{1}{{\left(1-4\cdot \frac{z_{i}^{2}}{D^{2}}\right)}^{\kappa }}\cdot \frac{2}{D}\cdot \int_{-D/2}^{D/2}{\left(1-4\cdot \frac{z_{i}^{2}}{D^{2}}\right)}^{\kappa }\cdot L_{i}.dz\;,$$

where the parameter κ is dependent on the type of integration used (κ=0.5 for the Gauss–Legendre method and κ=0.6 for the OWICS method, considering circular cross-section conduits in both cases). The variable $L_{i}$ is the Lagrange polynomial integration, calculated as (14).

(14) $$L_{i}\left(z\right)=\prod_{\begin{array}{c} j=1\\ j\neq i \end{array}\;}^{N}\frac{z-z_{j}}{z_{i}-z_{j}}\;.$$

**Figure 6:**
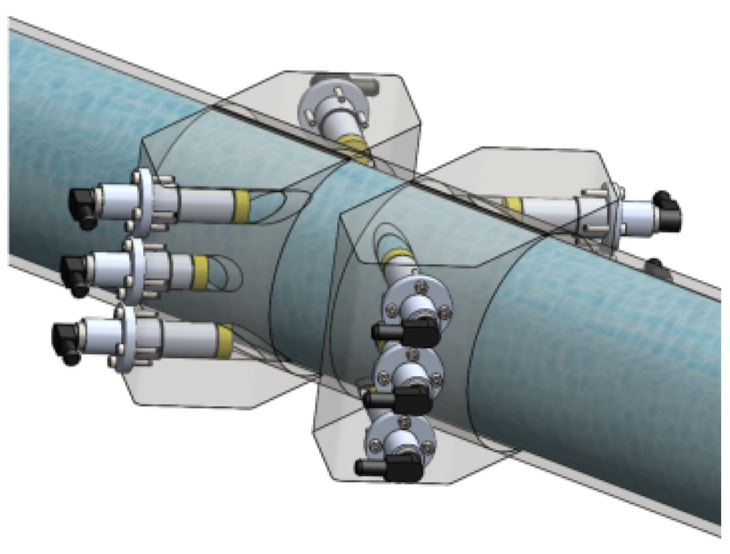
Ultrasonic sensors forming two planes of three trajectories.

The developed system was based on a measurement configuration using six acoustic trajectories introduced in a circular spool, as presented in Figure 6. The acoustic trajectories are distributed in two crossed measurement planes (*A* and *B*), containing three trajectories in each plane. Therefore, the flow rate for a measurement plan (*A* or *B*) is determined from Equation (11) and results in (15). Furthermore, each sensor forms an angle of $\varphi_{i}=45^{\circ }$ with the conduit wall.

(15) $$Q_{A|B}=\frac{D}{2}\cdot \sum_{i=1}^{3}w_{i}\cdot {\bar{v}}_{ax,i}\left(z_{i}\right)\cdot L_{i}\cdot \sin\left(\varphi_{i}\right)\;.$$

Using (15) for both planes *A* and *B*, the resultant flow rate can be obtained as the average (16).

(16) $$Q_{v}=\frac{Q_{A}+Q_{B}}{2}\;.$$

Each of the two planes has a trajectory positioned at the center of the circular spool and two other trajectories equidistant from the center, forming the positions $z_{1}=-0.707107\cdot D/2$, $z_{2}=0$ and $z_{3}=+0.707107\cdot D/2$. For each trajectory, based on Equations (13) and (14), the respective weights $w_{i}$ are presented in Table 2, either considering Gauss–Jacobi or OWICS integration methods.

Table 3 presents the measured lengths ($L_{i}$) of the trajectories for either planes *A* and *B*. Ideally, the lengths $L_{1}$ and $L_{3}$ of both planes should have the same value, as well as the lengths $L_{2}$ of both planes. However, due to a not so precise drilling, there are these 1 mm discrepancies.

#### 2.3.3. Evaluation Setup

**Figure 7:**
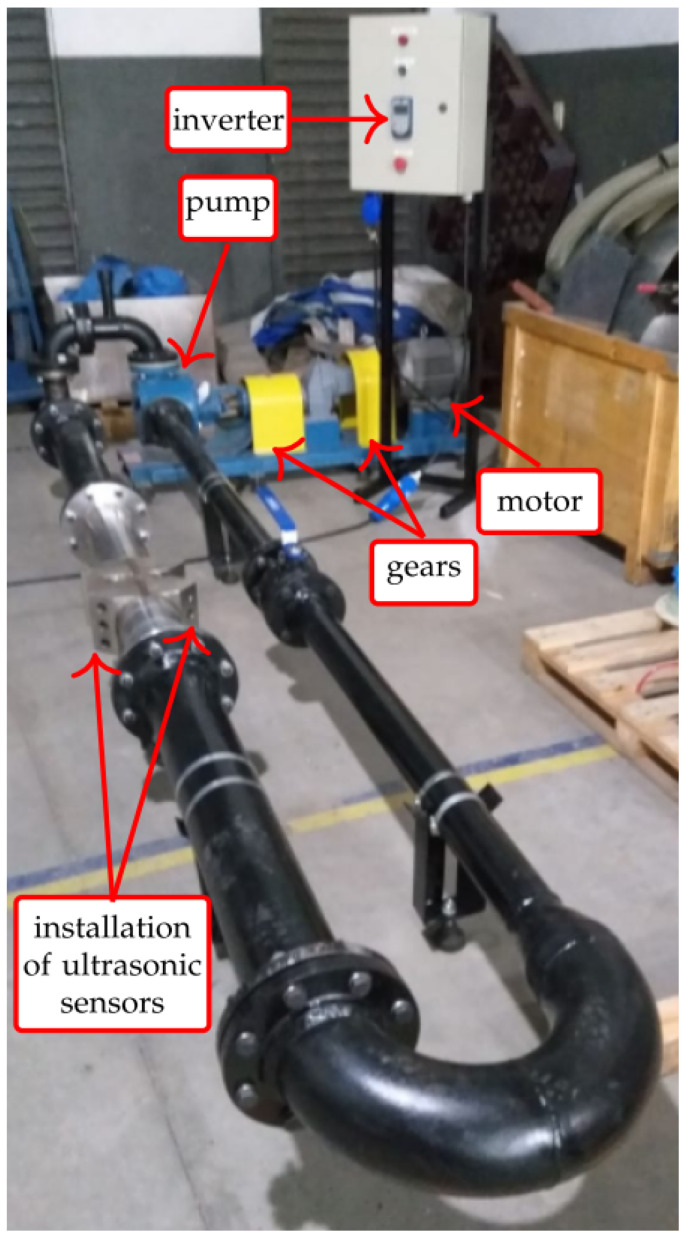
Evaluation setup for the ultrasonic sensors.

In order to evaluate the ultrasonic system, a reduced scale flow setup has been assembled, as presented in Figure 7. This setup is formed by a pump driven by an electric motor (whose speed is controlled by an inverter) and a closed loop hydraulic system.

With the setup of Figure 7, it was possible to vary the flow of the hydraulic circuit by controlling the rotation of the motor that drives the pump. In total, five motor speeds were used (0 RPM, 450 RPM, 500 RPM, 600 RPM, and 700 RPM) in order to achieve a more stable flow in the system. The motor speeds are reduced to the pump using a gear train (of ratio 2.54).

Water has been used as the circulating fluid, as it is it is cheaper than oil and its physical–chemical parameters are well known in the literature. For each motor speed, 100 complete transit time measurements were acquired for each of the 12 trajectories. Means and standard deviations were calculated from the measurements taken, as well as the uncertainties associated with the measurement.

Evaluation of Flow Measurements

Table 4 presents the obtained flows (measured with the ultrasonic system) for each motor rotation (as well as the respective pump rotations).

Ideally, the flow measurements obtained with the proposed sensors should be validated using a certified flow measurement system. As this was not possible, the solution found was to compare the measured flows against the data sheet of the pump [53], which relates flow with pump rotation. From the data of Table 4, a linear regression is performed, resulting in the Equation (17).

(17) $$Q_{prototype}=6.91275\;\times 10^{-2}\;\times RPM-2.43041\;\times 10^{-1}\;,$$

where RPM is the motor rotation (in RPM) and $Q_{prototype}$ is the interpolated flow (in m${}^{3}$/h) measured with the prototype.

The manufacturer curve of the pump can be obtained in [53] and results in Equation (18).

(18) $$Q_{pump}=6.88505\;\times 10^{-2}\;\times RPM-2.32743\;\times 10^{-1}\;,$$

where $Q_{pump}$ is the reference flow (in m${}^{3}$/h) for a motor rotation RPM.

Based on Equations (17) and (18), for some values of motor rotation, the flows of the proposed system can be compared against the theoretical flows at the pump. This comparison is presented in Table 5.

Evaluation of Speed-of-Sound and Density Measurements

As the fluid used in the evaluation setup was water, and the fluid temperature at the moment of the tests was 19.40 ${}^{\circ }$C, and the value of speed of sound can be obtained as 1480.36 m/s [54] and the value of density can be obtained as 998.34 kg/m${}^{3}$ [55]. These theoretical values can be used in order to validate the measurements of speed of sound and density performed with the proposed prototype. These comparisons are presented in Table 6 and Table 7.

As observed in Table 6 and Table 7, the deviations are below 0.2%.

### 2.4. Estimation of Heating Value

The heating values of any organic compound are associated with the bonding energies between the atoms that form the chemical structure of the compound and, therefore, the character of the bonds [56]. However, the possibility of calculation of the heating value for petroleum-derived fuels with reasonable accuracy from elementary composition alone took many researchers to establish empirical correlations from their commonly measured characteristics [57,58]. These correlations are often expressed in the form of linear combinations of the percentages by weight of the elements of the atoms of carbon (C), hydrogen (H), and oxygen (O), and sometimes expanded to sulfur (S) and nitrogen (N). The reason is that the main elements in the chemical composition of fossil fuels are, in fact, limited to C, H, O, N, and S in their organic part [58]. Furthermore, the contaminants present in the oils have a great influence on the efficiency of their combustion. Water is the contaminant that has the greatest influence on HHV and LHV levels, where the greater the amount of water, the lower the values of the two factors will be. Furthermore, sulfur and ashes are two other important contaminants.

The standard ASTM D4868 [13] presents an empirical method to estimate the HHV and the LHV based on the fuel density and the amounts of water, sulfur, and ashes. It is described by Equations (19) and (20).

(19) $$\begin{array}{ccc} HHV{|}_{ASTM\;D4868} & = & \left(51.916-8.792\cdot 10^{-6}\cdot \rho^{2}\right)\cdot \left[1-\left(f_{water}+f_{sulfur}+f_{ashes}\right)\right]\\ & + & 9.420\cdot f_{sulfur}\;, \end{array}$$

where ρ is the density of the fuel at 15 ${}^{\circ }$C (in kg/m${}^{3}$), obtained from a laboratory test. $f_{water}$ is the water content (in V/V), $f_{sulfur}$ is the sulfur content (in V/V), and $f_{ash}$ is the ash content (in V/V).

(20) $$\begin{array}{ccc} LHV{|}_{ASTM\;D4868} & = & \left(46.423-8.792\cdot 10^{-6}\cdot \rho^{2}+3.170\cdot 10^{-3}\cdot \rho \right)\cdot \left[1-\left(f_{water}+f_{sulfur}+f_{ashes}\right)\right]\\ & + & 9.420\cdot f_{sulfur}-2.449\cdot fwater\;. \end{array}$$

The amount of sulfur can be obtained through laboratory analysis using technologies such as ultraviolet fluorescence, non-dispersive infrared, or X-ray fluorescence spectroscopy [59]. These technologies measure the sulfur content at the molecular level and are very specific and difficult to use online. The same can be said for the ash content. Hence, this work considers only the water content.

The water content is obtained online (with the sensor and method presented in Section 2.2). Furthermore, the density is obtained online (with the sensor and method presented in Section 2.3). These two are applied in simplified versions of Equations (19) and (20), without considering the sulfur and ashes contents, resulting in Equations (21) and (22).

(21) $$HHV=\left(51.916-8.792\cdot 10^{-6}\cdot \rho_{0}^{2}\right)\cdot \left(1-fwater\right)\;,$$

(22) $$LHV=\left(46.423-8.792\cdot 10^{-6}\cdot \rho_{0}^{2}+3.170\cdot 10^{-3}\cdot \rho_{0}\right)\cdot \left(1-fwater\right)-2.449\cdot fwater\;,$$

where $\rho_{0}$ is the density of the fuel compensated in relation to the amount of water using Equation (23).

(23) $$\rho_{0}=\frac{\rho_{u}-\rho_{w}\cdot f_{water}}{1-f_{water}}\;,$$

where $\rho_{u}$ is the density of the fuel, as obtained with the ultrasonic sensor of Section 2.3. $\rho_{w}$ is the density of water.

## 3. Results and Discussion

**Figure 8:**
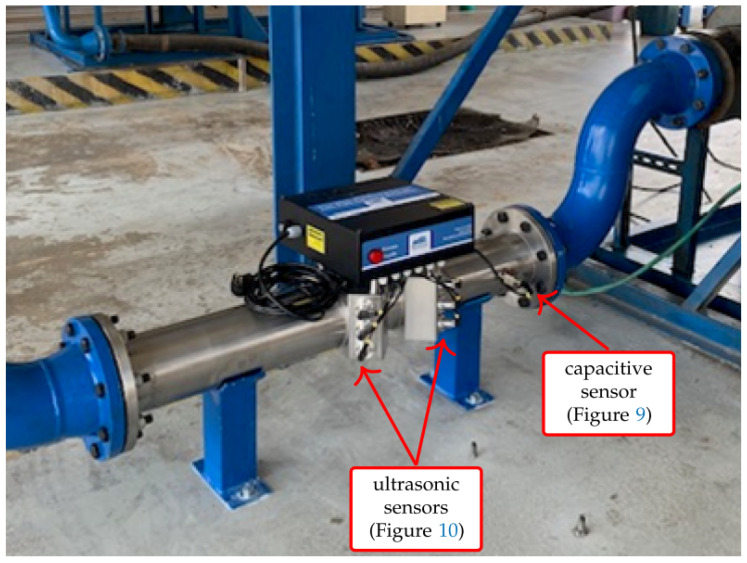
Proposed system installed at the fuel input of a Brazilian TPP.

The proposed system has been installed in a Brazilian TPP. Figure 8 presents a photograph of the setup, installed in a section of the pipeline between the TPP fuel storage and the point where the tank truck unloads the fuel oil. The system is composed of one capacitive sensor and six pairs of ultrasonic sensors, as described in Section 2.

**Figure 9:**
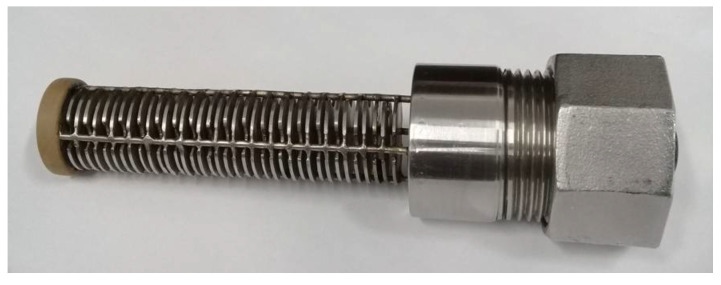
Proposed capacitive sensor for determination of water content.

Figure 9 presents a photograph of the proposed capacitive sensor for determination of water content, as described in Section 2.2.

**Figure 10:**
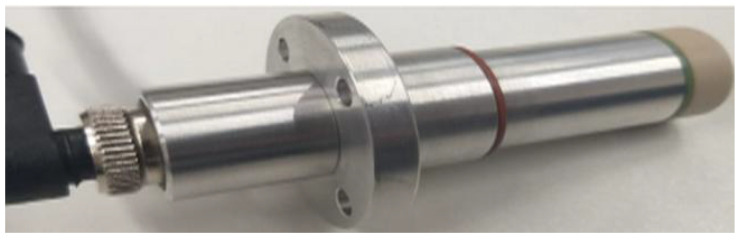
Proposed ultrasonic sensor for determination of density and flow.

Figure 10 presents a photograph of the proposed ultrasonic sensor for determination of density and flow, as described in Section 2.3.

The following results were obtained at three distinct operations of loading, with three different tank trucks. These tests were carried out at different periods due to the requirement of the plant itself for energy generation. During these periods, the measurement system was turned on and followed the supply process carried out by TPP. General loading information is presented in Table 8.

The prototype was configured to generate information in a 20-s update cycle at the time of loading. In these 20 s, acquisitions of signals and data processing were performed. In case of the ultrasonic system (which is responsible for measurement of flow measurement, sound velocity and, consequently, density), 600 UP and DOWN transit time samples were acquired for each acoustic trajectory, and from these measurements, the corresponding calculations were performed. Therefore, each 20 s update presents data for an average of 600 samples of the ultrasonic system. In case of the capacitive system, 5000 capacitance measurement samples were acquired per update cycle that are taken into account to calculate the water content of the emulsion.

The uncertainties presented in the graphs that follows are defined according to the guidelines given in reference [60]. In the case of the ultrasonic system process, the uncertainties are correlated to the measurement of transit times and take into account their mean, standard deviation, and number of samples for each update cycle. The other uncertainties (path length, angle, etc.) related to uncertainty propagation are static, as they involve measurements based on calibrated equipment. The same principle is used in the capacitive system, where the capacitance uncertainty is estimated by the mean, standard deviation, and number of samples of measurements performed in each update cycle. Uncertainty propagation for the other quantities, density, and LHV use the concept of uncertainty propagation [61].

### 3.1. Flow

**Figure 11:**
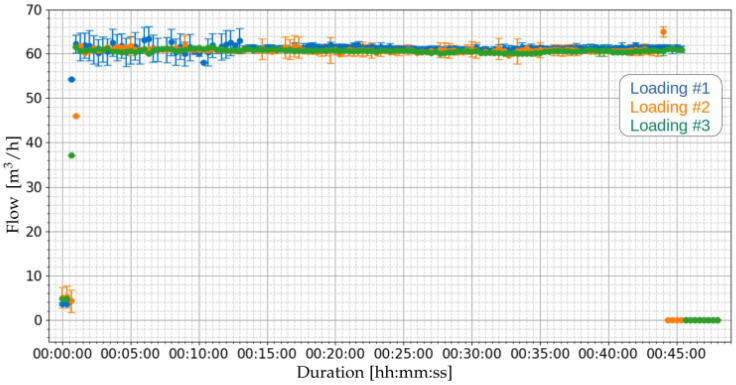
Measurement of flow using the proposed ultrasonic sensor of Section 2.3 for the three loading operations of Table 8.

**Figure 12:**
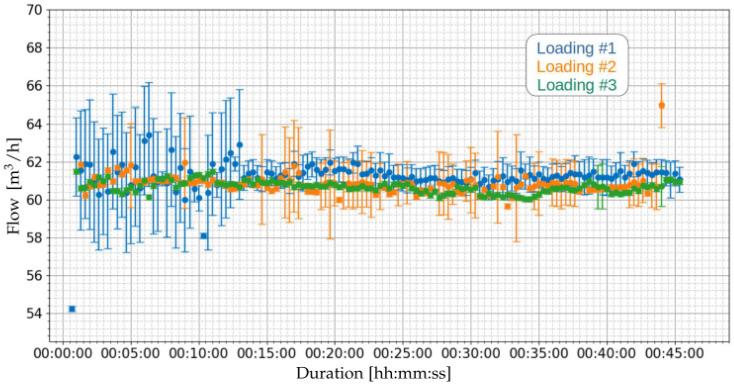
Zoomed view on the measurements of flow, in order to better visualize the uncertainties.

Figure 11 and Figure 12 present the results of the flow measurements performed for the loading operations of Table 8. Figure 11 presents the general perspective of the three loading operations. It can be noted that the flow measurements of the three operations are very close over time.

Figure 12 presents a zoom in the region of Figure 11 where the loading is actually happening, in order to visualize more clearly the calculated uncertainties. It is possible to observe that some samples present high levels of uncertainty. For the first loading (blue color) it is observed that the uncertainty levels at the beginning of the loading (from the start of the procedure until near the 15 min mark) had some samples with large levels of uncertainty, while the remaining samples had lower levels. These variations in the uncertainties are related to the dispersion of transit time differences (Equation (12)) with high standard deviations and, consequently, higher levels of uncertainty in the flow.

Table 9 presents the summary of the flow measurements of the three loading operations of Table 8. As noted, the average values of the means are close to each other and have similar standard deviations from the mean. Concerning the uncertainties, it can be seen that the maximum uncertainty observed between loading operations was 6.74%. Variations in uncertainties are related to the standard deviation of the difference in transit time ($\Delta_{t}=t_{up}-t_{down}$) of the trajectories.

The average fluid temperatures during the loading operations were 17.75 ${}^{\circ }$C, 19.58 ${}^{\circ }$C, and 21.55 ${}^{\circ }$C, for loading operations 1, 2, and 3, respectively.

### 3.2. Capacitance and Water Content

**Figure 13:**
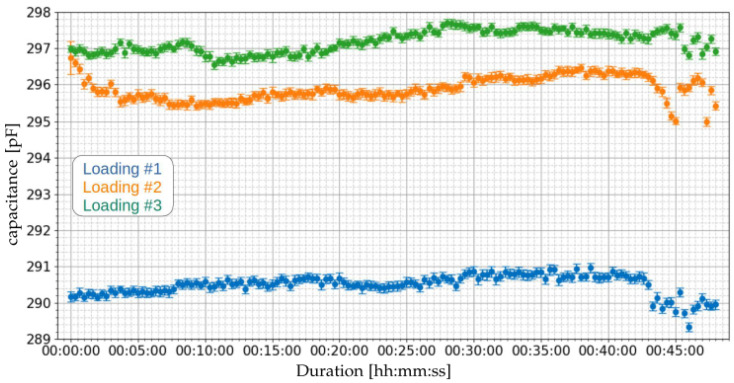
Measurement of capacitance using the proposed capacitive system of Section 2.2 for the three loading operations of Table 8.

Figure 13 presents the results of the flow measurements performed for the loading operations of Table 8. Table 10 presents the summary of the capacitance measurements of the three loading operations.

**Figure 14:**
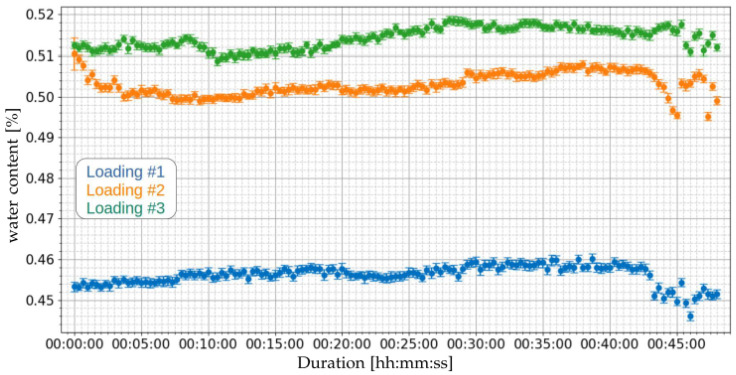
Measurement of water content using the proposed capacitive system of Section 2.2 for the three loading operations of Table 8.

Based on the measured capacitances, applying Equation (1), the water content for the loading operations of Table 8 can be obtained. Figure 14 presents the estimated water contents. Table 11 presents the summary of the water content measurements of the three loading operations. It can be noted that the average water contents were 0.456%, 0.503%, and 0.514%, for loading operations 1, 2, and 3, respectively.

The results obtained using the capacitive sensor should have, ideally, been compared against a laboratory test. However, the only laboratory results available had been obtained almost three months before the installation of the prototype, but they are used here, in order to give an idea of how close the proposed system compares to a laboratory analysis. The complete laboratory results are presented in the Appendix A. The results obtained with the prototype indicate around 0.5% of water content. The laboratory results indicated 0.05%. This a (somehow) large discrepancy—although both values are well below the limit of 2% determined by the Brazilian National Agency of Petroleum, Natural Gas and Biofuels (ANP) [36].

### 3.3. Speed of Sound and Density

**Figure 15:**
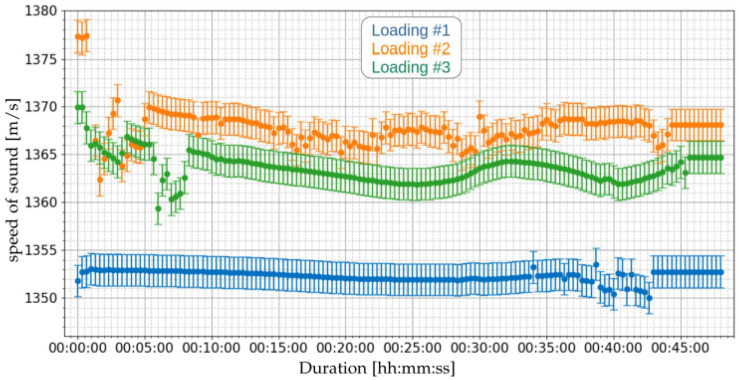
Measurement of speed of sound using the proposed ultrasonic system of Section 2.3 for the three loading operations of Table 8.

Figure 15 presents the results of the online measurements of the speed of sound at the emulsion, performed for the loading operations of Table 8. Table 12 presents the summary of the speed of sound measurements of the three loading operations.

**Figure 16:**
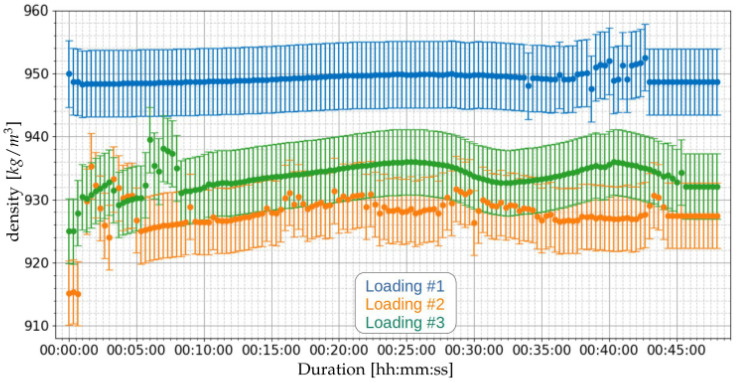
Measurement of density using the proposed ultrasonic system of Section 2.3 for the three loading operations of Table 8.

Based on the measured speeds of sound, applying Equation (23), the densities for the loading operations of Table 8 can be obtained. Figure 16 presents the estimated densities. Table 13 presents the summary of the density measurements of the three loading operations.

The results obtained with the prototype indicate average density values from 928.2 to 949.2 kg/m${}^{3}$. The laboratory results of the Appendix A indicated 927.7 kg/m${}^{3}$. This indicates a maximum discrepancy of 2.3%, although the laboratory tests have been performed three months before the installation of the prototype.

### 3.4. Heating Value

**Figure 17:**
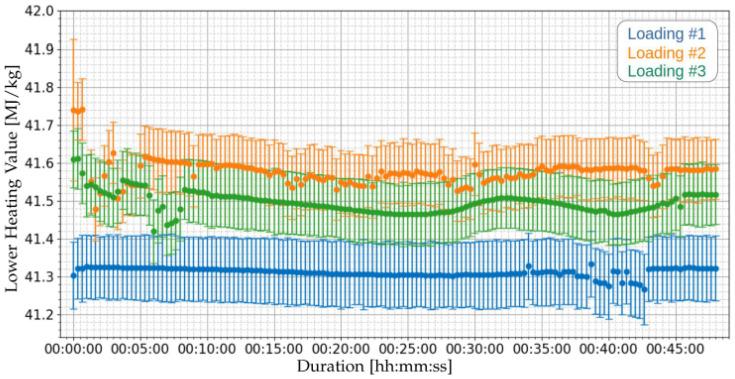
Measurement of Lower Heating Value using both the proposed capacitive system of Section 2.2 and the proposed ultrasonic system of Section 2.3 for the three loading operations of Table 8.

Using the measured values of the water content (data from Figure 14) and the density (data from Figure 16), the lower heating value can be estimated online, as presented in Figure 17.

Table 14 presents the summary of the LHV measurements of the three loading operations. It can be observed that the calculated uncertainties had values below 0.25%.

The results obtained with prototype indicate average LHV values from 41.31 to 41.57 MJ/kg. The laboratory results of the Appendix A indicate 41.59 MJ/kg. This indicates a discrepancy below 1%, although the laboratory tests have been performed three months before the installation of the prototype.

## 4. Conclusions

This paper presented a hybrid online monitoring system for the determination of fuel oil quality. It is proposed the association of two different measurement techniques (i.e., capacitive and ultrasonic techniques) and the correlation of their information in order to assess the quality parameters. The capacitive technique aims to measure the water content in the fuel oil, while the ultrasonic technique directly measures the fuel’s density and flow. The combination of information from both techniques serves to infer about other quality indicators and carry out calibration processes.

The estimation of water content through the capacitive technique is based on the fact that the dielectric properties of the fuel oil changes according to its water content. Hence, a capacitive sensor (composed of multiple metallic disks) is proposed in order to measure the capacitance of the emulsion (fuel oil with water contamination) that fills the spaces between the disks.

The estimation of density and flow through the ultrasonic technique is based on the propagation times of the ultrasonic pulses on the fuel oil. A system composed of an array of sensors is proposed in order to estimate the velocity of the pulses, even with the fuel in motion through the pipeline where the sensors are installed.

With the information of both the water content and the density, the heating value of the fuel oil can be estimated. The heating value is an important parameter that shows the quality of the fuel, which implies in the efficiency of the combustion cycle of the power generators. Usually, combustion-based power plants have to perform laboratory tests in order to determine the quality of their fuels. In these cases, samples are collected and sent to a laboratory for the analysis. With the proposed system, the analysis can be performed instantaneously, while the tank truck is loading the plant.

The proposed system, at the current stage of research, is an invasive procedure. As a future work, it is being studied a non invasive procedure, for both the capacitive and ultrasonic systems. Furthermore, as future work, it is planned to analyze the influence of flow conditions on the measurements of capacitance and the effects of the temperature on the density of the fuel oil. To date, due to intermittent loading operations at the TPP (few operations per month due to low demand), some long-term measurements, such as the evaluation of drift in the sensors, cannot be performed.

## Figures and Tables

**Table 1:** Calibration example of the capacitive sensor.

Karl Fischer Water Content	Measured Capacitance
0.19%	261.8 pF
0.40%	286.1 pF
1.01%	353.2 pF
2.20%	482.9 pF

**Table 2:** Weighting coefficients for a circular cross section conduit.

Trajectory *i*	Position $z_{i}/\left(D/2\right)$	$w_{i}$ for Gauss–Jacobi	$w_{i}$ for OWICS
1	−0.707107	0.555360	0.546150
2	0	0.785398	0.792715
3	+0.707107	0.555360	0.546150

**Table 3:** Lengths of the acoustic trajectories.

Trajectory *i*	Length $L_{i}$ for Plane A	Length $L_{i}$ for Plane B
1	162.26 mm	163.54 mm
2	203.00 mm	202.83 mm
3	163.05 mm	162.93 mm

**Table 4:** Relation between flow and motor/pump angular speed.

Motor Rotation	Pump Rotation	Flow
0 RPM	0.00 RPM	0.00 m${}^{3}$/h
450 RPM	117.17 RPM	11.47 m${}^{3}$/h
500 RPM	196.85 RPM	13.19 m${}^{3}$/h
600 RPM	236.22 RPM	16.07 m${}^{3}$/h
700 RPM	275.59 RPM	19.29 m${}^{3}$/h

**Table 5:** Comparison between the theoretical flows against the interpolated flows of the prototype.

Motor Rotation	$Q_{\mathrm{pump}}$	$Q_{\mathrm{prototype}}$	Deviation
400 RPM	27.41 m${}^{3}$/h	27.31 m${}^{3}$/h	0.37%
500 RPM	34.32 m${}^{3}$/h	34.19 m${}^{3}$/h	0.37%
600 RPM	41.23 m${}^{3}$/h	41.08 m${}^{3}$/h	0.38%
700 RPM	48.15 m${}^{3}$/h	47.96 m${}^{3}$/h	0.38%

**Table 6:** Validation of measurements of speed of sound of water circulating in the evaluation setup at 19.40 ${}^{\circ }$C, whose theoretical value is 1480.36 m/s [54].

Motor Rotation	Speed of Sound	Deviation
0 RPM	1479.01 m${}^{3}$/h	0.091%
450 RPM	1480.52 m${}^{3}$/h	0.011%
500 RPM	1480.31 m${}^{3}$/h	0.004%
600 RPM	1480.73 m${}^{3}$/h	0.025%
700 RPM	1481.10 m${}^{3}$/h	0.034%

**Table 7:** Validation of measurements of density of water circulating in the evaluation setup at 19.40 ${}^{\circ }$C, whose theoretical value is 998.34 kg/m${}^{3}$ [55].

Motor Rotation	Density	Deviation
0 RPM	1000.16 kg/m${}^{3}$	0.183%
450 RPM	998.13 kg/m${}^{3}$	0.021%
500 RPM	998.41 kg/m${}^{3}$	0.007%
600 RPM	997.84 kg/m${}^{3}$	0.050%
700 RPM	997.35 kg/m${}^{3}$	0.069%

**Table 8:** Recorded loading operations at the TPP.

Loading	Date	Time	Duration
1	9 June 2021	10:50–11:35	45${}^{\prime }$20${}^{\prime \prime }$
2	30 June 2021	10:52–11:36	44${}^{\prime }$00${}^{\prime \prime }$
3	30 June 2021	12:05–12:50	45${}^{\prime }$20${}^{\prime \prime }$

**Table 9:** Summary of the flow measurements of the three loading operations of Table 8.

Loading	Average of the Means	Standard Deviation of the Means	Minimum Uncertainty	Maximum Uncertainty
1	61.3 m${}^{3}$/h	0.3 m${}^{3}$/h	0.23%	6.74%
2	60.8 m${}^{3}$/h	0.3 m${}^{3}$/h	0.23%	5.93%
3	60.7 m${}^{3}$/h	0.3 m${}^{3}$/h	0.23%	1.31%

**Table 10:** Summary of the capacitance measurements of the three loading operations of Table 8.

Loading	Average of the Means	Standard Deviation of the Means	Minimum Uncertainty	Maximum Uncertainty
1	290.5 pF	0.3 pF	0.032%	0.054%
2	297.4 pF	0.5 pF	0.028%	0.152%
3	295.9 pF	0.3 pF	0.024%	0.048%

**Table 11:** Summary of the water content measurements of the three loading operations of Table 8.

Loading	Average of the Means	Standard Deviation of the Means	Minimum Uncertainty	Maximum Uncertainty
1	0.456%	0.002%	0.18%	0.30%
2	0.503%	0.003%	0.14%	0.77%
3	0.514%	0.003%	0.12%	0.25%

**Table 12:** Summary of the speed of sound measurements of the three loading operations of Table 8.

Loading	Average of the Means	Standard deviation of the Means	Minimum Uncertainty	Maximum Uncertainty
1	1352.4 m/s	0.5 m/s	0.122%	0.122%
2	1367.6 m/s	1.2 m/s	0.121%	0.142%
3	1363.5 m/s	1.3 m/s	0.121%	0.122%

**Table 13:** Summary of the density measurements of the three loading operations of Table 8.

Loading	Average of the Means	Standard Deviation of the Means	Minimum Uncertainty	Maximum Uncertainty
1	949.2 kg/m${}^{3}$	0.6 kg/m${}^{3}$	0.556%	0.556%
2	928.2 kg/m${}^{3}$	1.6 kg/m${}^{3}$	0.556%	0.575%
3	933.8 kg/m${}^{3}$	1.7 kg/m${}^{3}$	0.556%	0.566%

**Table 14:** Summary of the Lower Heating Values measurements of the three loading operations of Table 8.

Loading	Average of the Means	Standard Deviation of the Means	Minimum Uncertainty	Maximum Uncertainty
1	41.31 MJ/kg	0.01 MJ/kg	0.192%	0.222%
2	41.57 MJ/kg	0.02 MJ/kg	0.181%	0.210%
3	41.49 MJ/kg	0.02 MJ/kg	0.180%	0.211%

## Data Availability

Not applicable.

## Abbreviations

The following abbreviations are used in this manuscript:

FO	Fuel Oil
GHC	Gross Heat of Combustion
HFO	Heavy Fuel Oil
HHV	Higher Heating Value
LFO	Light Fuel Oil
LHV	Lower Heating Value
NHC	Net Heat of Combustion
TPP	Thermal Power Plant

## Appendix A. Laboratory Tests Performed on a Sample of HFO

Table A1 presents an excerpt of the results sheet of a laboratory analysis performed on a sample of HFO of the same TPP where the prototype has been installed. The test, however, has been performed some months before the installation of the system.

**Table A1:** Results of laboratory tests performed on a sample of HFO (from the results sheet provided by the Brazilian laboratory Intertek).

Methods	Tests	Results	Units
ASTM D1298	density (20/4 ${}^{\circ }$C)	927.7	kg/m${}^{3}$
ASTM D95	water by distillation	0.05	% of volume
ASTM D4868	lower heating value	41.59	kJ/kg
